# Monitoring the growth dynamics of *Tetragenococcus halophilus* strains in lupine moromi fermentation using a multiplex-PCR system

**DOI:** 10.1186/s13104-023-06406-y

**Published:** 2023-06-22

**Authors:** Tobias Link, Matthias A. Ehrmann

**Affiliations:** Lehrstuhl für Mikrobiologie, Technische Universität München, 85354 Freising, Germany

**Keywords:** Tetragenococcus halophilus, Strain dynamic changes, Lupine moromi, Starter culture, Multiplex PCR

## Abstract

**Objective:**

The microbiota of a seasoning sauce fermentation process is usually complex and includes multiple species and even various strains of one species. Moreover, composition and cell numbers of individual strains vary over the course of the entire fermentation. This study demonstrates the applicability of a multiplex PCR system to monitor growth dynamics of *Tetragenococcus (T.) halophilus* strains in order to evaluate their performance and help to select the most competitive starter strain.

**Results:**

In a previous study we isolated *T. halophilus* strains from multiple lupine moromi fermentation processes and characterized them. In this study we wanted to monitor the growth dynamics of these strains in a competitive lupine moromi model fermentation process using a multiplex PCR system. Therefore, pasteurized lupine koji was inoculated with eight different *T. halophilus* strains, six from lupine moromi, one from an experimental buckwheat moromi fermentation process and the type strain DSM 20,339^T^, to create the inoculated lupine moromi pilot scale fermentation process. With the multiplex PCR system, we could detect that all strains could grow in lupine moromi but, that TMW 2.2254 and TMW 2.2264 outperformed all other strains. Both strains dominated the fermentation after three weeks with cell counts between 4 × 10^6^ to 4 × 10^7^ CFU/mL for TMW 2.2254 and 1 × 10^7^ to 5 × 10^7^ CFU/mL for TMW 2.2264. The pH dropped to value below 5 within the first 7 days, the selection of these strains might be related to their acid tolerance.

**Supplementary Information:**

The online version contains supplementary material available at 10.1186/s13104-023-06406-y.

## Introduction

The fermentation of vegetables, meat or fish is a common way to preserve or improve the quality of these foods [1–3]. Many fermented foods originating from Asia include high concentrations of sodium chloride to prevent spoilage, e.g. soy sauce [4]. Recently, a seasoning sauce made from lupine beans instead of soybeans was introduced to the market and the microbiota was characterized [5]. One of major species commonly isolated from this lupine bean (moromi) fermentation, soy sauce, Korean soy pastes, salted fish or fish sauce is *T. halophilus* [6–9]. This species is well known for its ability to grow and form acids in cultivations broths containing high concentrations of sodium chloride [10, 11]. Within the fermentation process, this species contributes to the flavor by the formation of organic acids and various volatile compounds [12, 13]. However, despite the use of sodium chloride, biogenic amines such as histamine can be detected in high concentrations in some fermentation processes and be traced back to the growth of some strains of *T. halophilus* [14, 15]. To prevent the formation of biogenic amines and standardize the fermentation process, specific strains are used as starter culture [16–18]. The fact that multiple strains can coexist in the same moromi fermentation at the same time, makes the development of defined single strain starter cultures difficult [19]. To monitor the strain dynamics within food fermentation process multiple systems can be used, either MALDI-TOF MS using a biotyper, RT-qPCR with strain specific primers or length polymorphism PCR [20–22]. However, some of these systems cannot be directly used for identifying *T. halophilus* strains, as the MALDI-TOF MS biotyper system does not allow strain level resolution and only a few strains encode for a CRISPR system [6, 23, 24]. Previously, a system to monitor the growth dynamics of *T. halophilus* within a fish fermentation process has been published using RT-qPCR, but with this system it was not possible to distinguish individual strains [25]. To distinguish between strains of *T. halophilus* Random Amplified Polymorphic DNA PCR (RAPD-PCR) can be used, but this technique requires gDNA isolation and therefore is not suitable for a higher throughput [26].

The strains used in this study have been genomically characterized and could up to this point only be distinguished by Random Amplified Polymorphic DNA PCR (RAPD-PCR) using our M13V primer, by their carbohydrate utilization or by whole genome comparison using ANI values (Figure S1) [6, 27]. To develop a defined single strain starter culture, a strain that can dominate and thereby control the fate of the fermentation process must be identified within a given set of strains. Therefore, we inoculated eight strains from three origins into a small sized lupine moromi and monitored the strain dynamics using this multiplex PCR system to identify every strain on a colony basis.

## Main text

### Methods

#### Strains and cultivation conditions

The strains used in this study were isolated from three different sources, lupine moromi, buckwheat moromi and salted anchovies. The lupine moromi was prepared from the beans of *Lupinus angustifolius* grown in Germany as described in [5]. The buckwheat moromi was prepared from buckwheat grown in Germany and prepared like the lupine moromi with the exception that 2% lupine protein powder was added. Six of the strains, namely TMW 2.2254, TMW 2.2256, TMW 2.2257, TMW 2.2263, TMW 2.2264 and TMW 2.2266 were isolated from lupine moromi. TMW 2.2260 was isolated from buckwheat moromi and DSM 20,339^T^ was originally isolated from salted anchovies.

To prepare precultures for the experiment, MRS medium (composed of 10 g/L casein peptone, 10 g/L meat extract, 5 g/L yeast extract, 20 g/L glucose, 1 g/L Tween80, 2 g/L di-potassium hydrogen phosphate trihydrate, 5 g/L sodium acetate trihydrate, 2 g/L di-ammonium hydrogen citrate, 0.2 g/L magnesium sulfate heptahydrate, 0.05 g/L manganese sulfate monohydrate) containing 5% (w/v) sodium chloride was inoculated with single colonies of each strain in 50 mL conical tubes and incubated at 30 °C for 48 h. Then, the OD_600nm_ was determined using a Novaspec Plus spectrometer (AK Kappenberg, Münster, Germany). Then, the correct volume of the precultures from every strain was transferred into fresh conical tubes that were required to create a 200 mL inoculation solution with an OD_600nm_ of 0.05. The cells were harvested by centrifugation at 12,000 x g for 15 min. The harvested cells were resuspended in 10 mL of sterile saline with 13.5% (w/v) sodium chloride using a vortex (Starlab International GmbH, Hamburg, Germany). The solutions of every strain were pooled into one big sterile flask and the flask was filled up to 200 mL with sterile saline with 13.5% (w/v) sodium chloride, creating an inoculation solution with cells of every strain.

#### Preparation of the small sized moromi

Lupine koji was prepared by soaking toasted and cracked lupine beans in water. Afterwards, the moist beans were inoculated with an *Aspergillus oryzae* and fermented for two days in an industrial fermentation tank at the Purvegan factory (Ramsen, Germany). The mature koji was then heated homogeneously to 80 °C for 15 min in an oven and then packaged and sealed using a vacuum packing machine. To prepare the moromi, 20 g of pasteurized lupine koji prepared at the Purvegan factory was filled in a sterile 50 mL conical tube (Sarstedt, Nümbrecht, Germany) and mixed with the inoculation solution. This solution contained 13.5% (w/v) sodium chloride and 2.6 × 10^6^ CFU/mL of total *T. halophilus* cells with every strain being in the range of 10^5^ CFU/mL. The tubes were filled to a volume of 50 mL. The closed conical tubes were kept at 25 °C for three weeks. For sampling the conical tubes were opened once a week under sterile conditions, mixed lightly with a sterile inoculation loop and 1 mL of the liquid phase was transferred into a fresh 1.5 mL Eppendorf tube.

#### Growth and pH determination

Growth of the *T. halophilus* cells within the moromi was determined by preparing serial dilutions of a sample from every triplicate in full-strength Ringer solution (Merck Millipore, Burlington, MA, USA) containing 5% (w/v) sodium chloride and subsequently plating out on MRS-Agar with 5% (w/v) sodium chloride. The plates were incubated at 30 °C in a sealed anaerobic jar containing an AnaeroGen™ bag (Thermofisher, Waltham, MA, USA). After 3 days, plates containing 20 to 200 colonies were counted and considered for the determination of the cell count. To measure the pH of the moromi, a sample transferred into a 1.5 mL test tube was centrifuged at 7,000 x g for 6 min and then diluted to a sodium chloride concentration of 5% (w/v). The pH was then measured using a 761 Calimatic pH meter (Knick GmbH & Co. KG, Berlin, Germany).

#### DNA isolation and whole genome sequencing

Genomic DNA (gDNA) of TMW 2.2260 was isolated and sequenced as previously described [6]. Briefly, genomic DNA of TMW 2.2260 was isolated using the E.Z.N.A Bacterial DNA-Kit (Omega bio-tek, Norcross, Georgia, USA) according to the manufacturer`s instructions. Then, the genomic DNA was sequenced by Eurofins Genomics (Konstanz, Germany) with an Illumina HiSeq. Previously sequenced genomes were taken from NCBI Database with following assembly accession numbers TMW 2.2254 (GCF_024137165.1), TMW 2.2256 (GCF_024137145.1), TMW 2.2257 (GCF_024137175.1), TMW 2.2263 (GCF_024137125.1), TMW 2.2264 (GCF_024137075.1), TMW 2.2266 (GCF_024137065.1) and type strain DSM 20,339^T^ (GCF_003841405.1).

#### Selection of strain specific regions and primer design

Strain specific regions were found using the automated process within the “Rapid identification of PCR primers for unique core sequences” (RUCS) version 1.0 program [28]. This was done by genomic comparison using the target strains as a “positive strain” and all the other strains as “negative strains”. Then, the program automatically designed primer pairs for these regions based on the standard settings with the maximum fragment size of 3 Kb. This procedure was done for all strains with the same settings. The strain specific primers used in this study can be found in Table S1. To avoid using primer that might form secondary structures, each primer was checked using the NetPrimer tool from Premier Biosoft (https://www.premierbiosoft.com/netprimer/). The Tm calculator of NEB (https://tmcalculator.neb.com/#!/main#!%2F) was used to ensure that the annealing temperature of every primer was the same and calculated with 55°C. During the development of this assay the annealing temperature was increased to 57°C to reduce unspecific bindings.

#### Strain identification ***via*** colony PCR

To perform colony PCR, 100 single colonies per replicate were picked using sterile toothpicks and smeared into sterile PCR tubes. Next, 25 µL of a PCR-Mix was added. The PCR-Mix consisted of 2.5 µL 10x standard buffer with MgCl_2_(MP Biomedicals, Eschwege, Germany), dNTPs at a final concentration of 200 µM, every primer at a final concentration of 31.25 nM (Eurofins Genomics, Ebersberg, Germany), 1.25 U of Taq polymerase (MP Biomedicals, Eschwege, Germany) and 21.25 µl of sterile 0.22 μm filtered H_2_O. An initial denaturation at 95 °C was done for 300 s to lyse the cells and denature the DNA. 25 amplification cycles were done in total each consisting of denaturation at 95 °C for 30 s followed by annealing at 57 °C for 30 s and elongation at 72 °C for 150 s. The final elongation was also done at 72 °C for 300 s. The PCR was carried out with a Mastercycler gradient (Eppendorf, Hamburg, Germany).

## Results and discussion

### Monitoring the strain compositional changes in the small sized lupine moromi

**Fig. 1:**
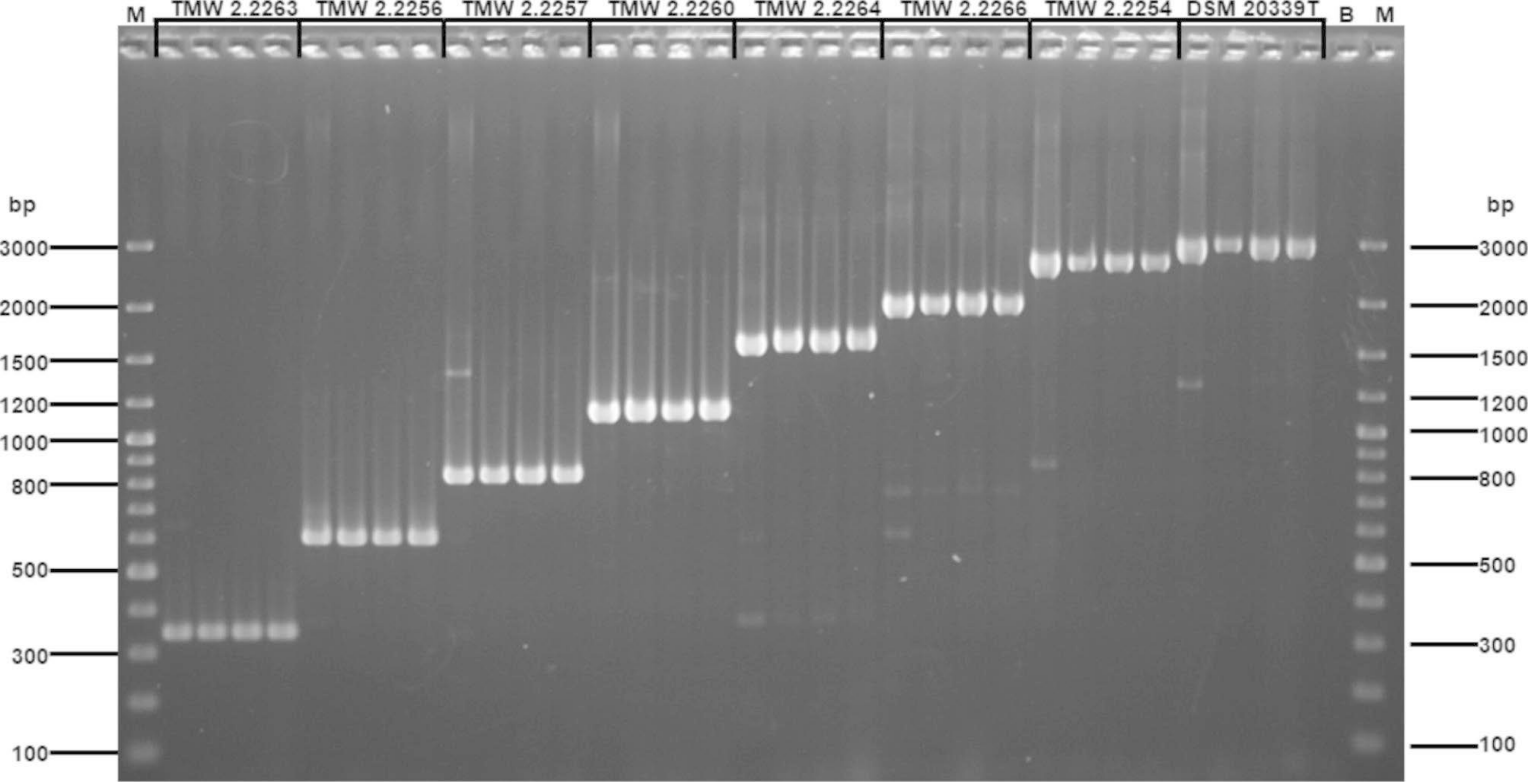
Amplified DNA fragments using the designed primer set. M = Generuler 100 bp, (Thermofisher Scientific, Waltham, MA, USA); B = Buffer control. Strain specific bands were generated using either gDNA (most left lane of every strain) or 3 individual single colonies of the respective strain. Expected fragment sizes: TMW 2.2254 = 2552 bp; TMW 2.2256 = 596 bp; TMW 2.2257 = 822 bp; TMW 2.2260 = 1119 bp; TMW 2.2263 = 333 bp; TMW 2.2264 = 1583 bp; TMW 2.2266 = 1961 bp; DSM 20339^T^ = 2882 bp. The picture was taken with a Gel Jet-imager system (Intas Science Imaging, Instruments GmbH, Göttingen, Germany) with the device software version 3.2.3.4089

The designed primer set enabled the identification of all eight strains (Fig. 1). Every strain could be clearly identified and separated by the length of the resulting product from the colony PCR. Only when using the pure high molecular gDNA as input unspecific bands appeared. However, as this primer-set was designed to identify strains *via* colony PCR, these were considered as negligible.

**Fig. 2:**
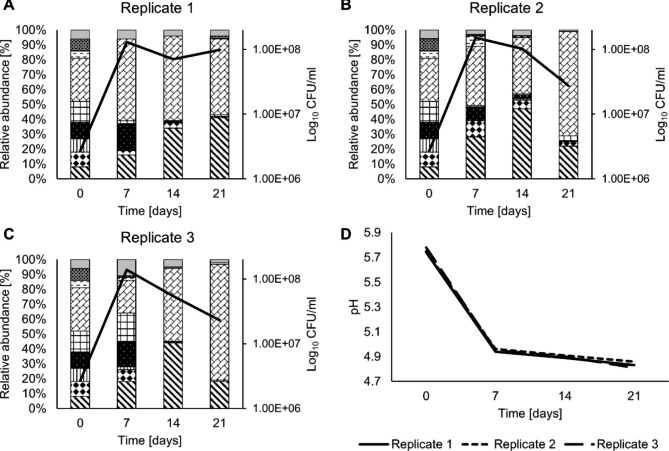
Monitoring of the strain composition and the pH development in a small sized lupine moromi. **A** = First pilot fermentation replicate; **B** = Second pilot fermentation replicate; **C** = Third pilot fermentation replicate; **D** = pH values of the three replicates over the course of 21 days. Legend in **A**, **B** and **C** starting from the bottom of every column: () = TMW 2.2254; () = TMW 2.2256; () = TMW 2.2257; () = TMW 2.2260; () = TMW 2.2263; () = TMW 2.2264; () = TMW 2.2266; () = DSM 20339^T^; gray = not clearly identifiable, meant that none or multiple bands occurred and this colony was not assigned to a specific strain. Black line in **A**, **B** and **C** represent the respective cell count in CFU/mL of the replicate at every sampling point

The strain dependent dynamics and the decrease of the pH value within each moromi replicate were monitored using the developed colony PCR primer set and a pH meter over the course of three weeks (Fig. 2, A-D). As all the inoculated strains can hypothetically grow in this environment, the total cell count increased from 2.68 × 10^6^ CFU/mL to at least 1 × 10^8^ CFU/mL in all replicates. The calculated cell counts based on the total cell counts and the distribution within 100 colonies can be found in Table S2. After two weeks the total cell count declined in all replicates with two replicates to 5.45 and 7.1 × 10^7^ CFU/mL and one replicate only merely decreased from 1.52 × 10^8^ to 1.03 × 10^8^ CFU/mL. This is most likely due to the fact that the pH was below 5 after the first week in all samples and thereby selects for strains that could survive under these conditions due to a higher acid tolerance (Fig. 2, D). After three weeks the cell counts per mL of all replicates were in the range of 10^7^ and the strain composition was comparable across all replicates (Fig. 2). The detection limit was 10^5^ CFU/mL for sample day 0, 14 and 21 and for sample day 7 the detection limit was at 10^6^ CFU/mL. The multiplex primer set enabled the detection of strain dependent increases or decreases within a lupine moromi over the course of three weeks e.g., TMW 2.2257, TMW 2.2266 and DSM 20,339^T^ performed significantly worse than TMW 2.2254 and TMW 2.2264 across all replicates (Fig. 2, A-C). This allows to further reduce the strains composition by not considering strains that are not able to outperform other strains in the desired environment. Growth and survival within the environment are important as by definition the starter strain must be able to outperform other bacteria, to ensure a safe, reliable and reproducible fermentation process. We hypothesize, that due to the low pH within the first week, some strains decrease in their cell counts below the detection limit as they are more susceptible to low pH (Fig. 2). As the arginine deiminase pathway (ADI) is one of the major pathways in *T. halophilus* contributing to the acid tolerance and is known to be highly upregulated in response to high sodium chloride concentrations, it would be expected that strains encoding a functional pathway are the dominate strains [29–31]. However, among the dominant strains only TMW 2.2264 encodes for a functional version of the ADI pathway, so this cannot be the sole reason (Fig. 2). Another reason might be the possession of at least one copy of an alpha galactosidase (α-gal), as TMW 2.2254 encodes for one α-gal and TMW 2.2264 encodes for two non-identical copies of α-gal [6]. Lupine beans are known to be rich in raffinose family oligosaccharides (RFOs), which can cause flatulencies in humans when consumed in greater quantities [32, 33]. Therefore, it might be beneficial to have a starter strain that can utilize these RFOs and thereby reducing the amount of RFOs in the final product. However, both hypotheses are not entirely proven with this study and more results are needed to underline the importance of either the α-gal or ADI in the lupine moromi.

In conclusion, we could prove that a multiplex PCR system can be used to track the strain dynamics within a small sized lupine moromi. As we designed it as a colony PCR protocol such a system can easily be adapted for a new set of strains and is applicable for high throughput. This approach then could help to easily select for starter strains for other fermented products.

### Limitations

The limitation of our study is that this primer set does only work for our inhouse strains and not for any other strains. The designed approach only allows tracking of *T. halophilus* but can easily be adopted. Furthermore, the genome sequence for all strains must be known as it is a required input for the RUCS software. Consequently, for each new set of strains, a new primer set must be designed. Further, our approach is not necessarily new, but is an improvement to previously developed RT-qPCR system [25] which allowed tracking of a *T. halophilus* strain in a fish sauce fermentation process but could not discriminate between strains.

## Electronic supplementary material

Below is the link to the electronic supplementary material.

Supplementary Material 1

Supplementary Material 2

## Data Availability

The published genome of TMW 2.2260 is available at the assembly accession number: within the BioProject: PRJNA640297.

## Abbreviations

PCR: Polymerase chain reaction
MALDI-TOF MS: Matrix-assisted laser desorption/ionization
RT-qPCR: Real-Time Quantitative Polymerase Chain Reaction
CRISPR: Clustered Regularly Interspaced Short Palindromic Repeats
