# Supplementary material for: Monitoring the growth dynamics of Tetragenococcus halophilus strains in lupine moromi fermentation using a multiplex-PCR system
Source: BMC Res Notes. 2023 Jun 22;16:115. doi: 10.1186/s13104-023-06406-y (PMC10288697; doi:10.1186/s13104-023-06406-y)
Supplement: Supplementary file 1 — Supplementary Material 1 [file 13104_2023_6406_MOESM1_ESM.docx]

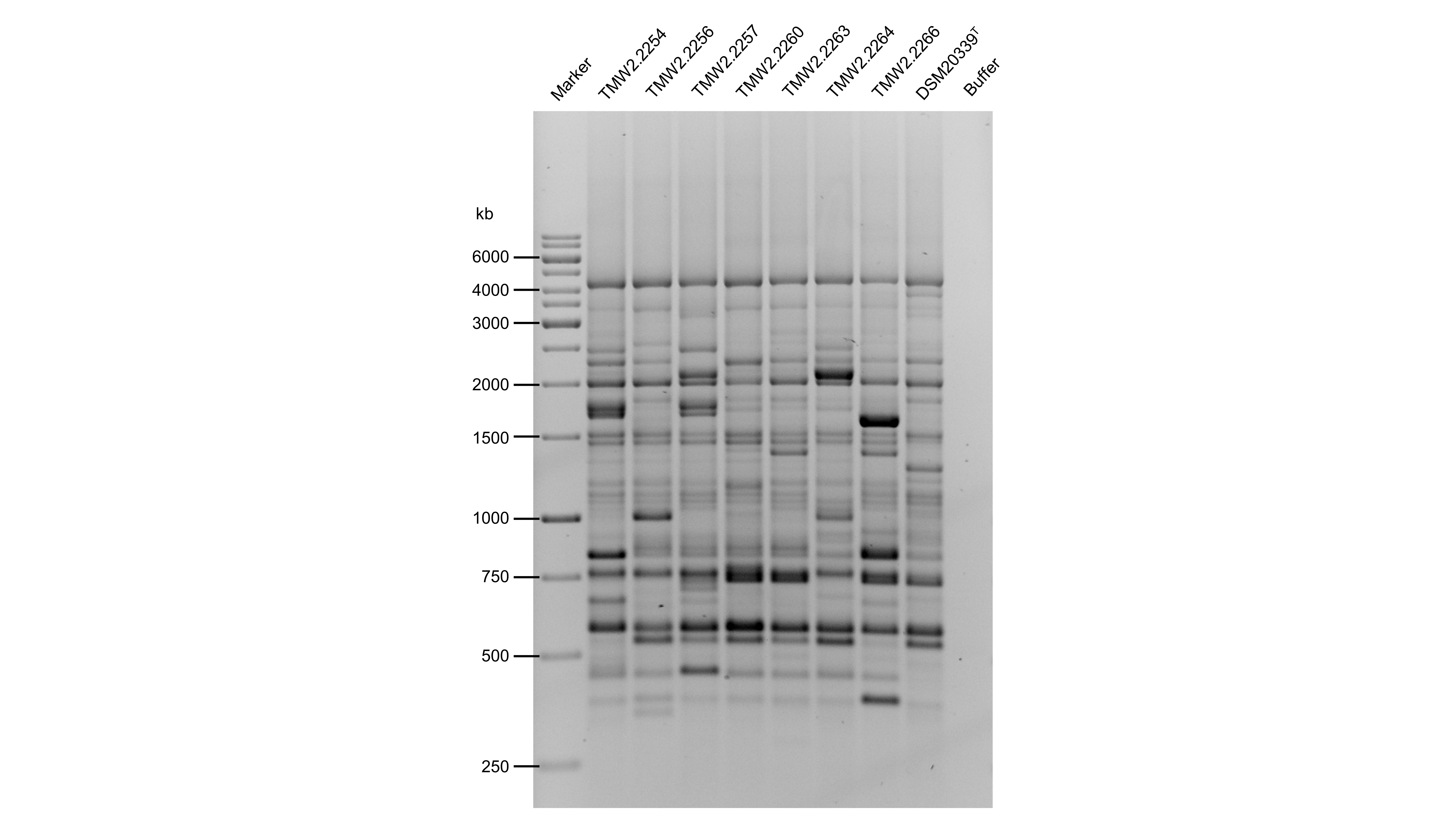


Figure S1: Identification of T. halophilus strains used in this study via strain specific RAPD-Fingerprint using the M13V [28] primer within a RAPD PCR. The picture was taken with a Gel Jet-imager system (Intas Science Imaging, Instruments GmbH, Göttingen, Germany) with the device software version 3.2.3.4089.
