# Supplementary material for: Monitoring the growth dynamics of Tetragenococcus halophilus strains in lupine moromi fermentation using a multiplex-PCR system
Source: BMC Res Notes. 2023 Jun 22;16:115. doi: 10.1186/s13104-023-06406-y (PMC10288697; doi:10.1186/s13104-023-06406-y)
Supplement: Supplementary file 2 — Supplementary Material 2 [file 13104_2023_6406_MOESM2_ESM.docx]

| Primer name | NCBI ORF and annotation | Sequence | Product Size | GC % | Reference |
| --- | --- | --- | --- | --- | --- |
| M13V | X | GTTTTCCCAGTCACGAC | X | 53 | [27] |
| TMW 2.2254 fwd | HV360_08825  PTS transporter subunit EIIC | CGTCCGGGTGTCTTAAAGTCA | 2552 bp | 52 | This study |
| TMW 2.2254 rev | HV360_08835  Gfo/Idh/MocA family oxidoreductase | AGCTCGCTATTTGGGTGGAAA |  | 48 | This study |
| TMW 2.2256 fwd | HXW74_05975  ethanolamine ammonia-lyase subunit EutB | AATCCCGACACAAACTAGCGT | 596 bp | 48 | This study |
| TMW 2.2256 rev | HXW74_05975  ethanolamine ammonia-lyase subunit EutB | GGACCGTAGCTGTTTCTTGGA |  | 52 | This study |
| TMW 2.2257 fwd | HXW75_03115  nucleoside hydrolase | CCTAAACCGTCTTCACCGTGA | 822 bp | 52 | This study |
| TMW 2.2257 rev | HXW75_03120  multidrug transporter | CTCCTCAAGGTCGTCAAAGCT |  | 52 | This study |
| TMW 2.2260 fwd | HXW78_09960  dapA, 4-hydroxy-tetrahydrodipicolinate synthase | AAGTGCATGTCCTTCCCCATT | 1119 bp | 48 | This study |
| TMW 2.2260 rev | HXW78_09965  PTS transporter subunit IIC | CGCTTGTGTGTTAGCTTTGCT |  | 48 | This study |
| TMW 2.2263 fwd | HXW81_02015  P27 family phage terminase small subunit | GAGCCAACTGTAGACGACACA | 333 bp | 52 | This study |
| TMW 2.2263 rev | HXW81_02020  terminase large subunit | CACCACGTCCCATTGTTAGGA |  | 52 | This study |
| TMW 2.2264 fwd | HXW82_01240  sulfatase | TGCCAGCTCGTCGAGATATTC | 1583 bp | 52 | This study |
| TMW 2.2264 rev | HXW82_01245  SPASM domain-containing protein | CCAATGTAGGCTCACCACCTT |  | 52 | This study |
| TMW 2.2266 fwd | HXW84_06150  transglycosylase SLT domain-containing protein | GTCAATGGCTTGGTGTTTCCC | 1961 bp | 52 | This study |
| TMW 2.2266 rev | HXW84_06150  transglycosylase SLT domain-containing protein | TTCCGTTGGCTGGATTCGTAA |  | 48 | This study |
| DSM 20339^T^ fwd | C7K42_05055  PTS trehalose transporter subunit IIC | CACCCGTTAACCCCATTACCA | 2882 bp | 52 | This study |
| DSM 20339^T^  rev | PXYA01000001 (1053559 -1053579) | AGCAGGTTTGTCGGCATAAGA |  | 48 | This study |

Table S1: Names, Sequences and the target region of the Primers used and designed in this study. The size of the resulting fragment when both primers bind to the correct sequence is indicated in column 4.

|  | Replicate 1 | | | | Replicate 2 | | | | Replicate 3 | | | |
| --- | --- | --- | --- | --- | --- | --- | --- | --- | --- | --- | --- | --- |
| **Sample day** | **0 Days** | **7 Days** | **14 Days** | **21 Days** | **0 Days** | **7 Days** | **14 Days** | **21 Days** | **0 Days** | **7 Days** | **14 Days** | **21 Days** |
| **TMW 2.2254** | **2.14E+05** | **2.08E+07** | **2.41E+07** | **4.04E+07** | **2.14E+05** | **4.24E+07** | **4.84E+07** | **6.05E+06** | **2.14E+05** | **2.49E+07** | **2.40E+07** | **4.14E+06** |
| **TMW 2.2256** | **2.68E+05** | **3.90E+06** | **2.84E+06** | **9.85E+05** | **2.68E+05** | **1.67E+07** | **6.18E+06** | **8.25E+05** | **2.68E+05** | **1.11E+07** | n.D | **2.30E+05** |
| **TMW 2.2257** | **2.41E+05** | n.D | n.D | n.D | **2.41E+05** | n.D | **1.03E+06** | n.D | **2.41E+05** | **2.77E+06** | n.D | n.D |
| **TMW 2.2260** | **2.95E+05** | **2.34E+07** | **7.10E+05** | n.D | **2.95E+05** | **1.36E+07** | **2.06E+06** | n.D | **2.95E+05** | **2.35E+07** | n.D | n.D |
| **TMW 2.2263** | **3.75E+05** | **2.60E+06** | n.D | **9.85E+05** | **3.75E+05** | **1.52E+06** | **1.03E+06** | **1.10E+06** | **3.75E+05** | **2.63E+07** | **5.45E+05** | n.D |
| **TMW 2.2264** | **7.77E+05** | **7.15E+07** | **4.05E+07** | **5.02E+07** | **7.77E+05** | **6.06E+07** | **3.91E+07** | **1.93E+07** | **7.77E+05** | **3.05E+07** | **2.67E+07** | **1.79E+07** |
| **TMW 2.2266** | **1.34E+05** | n.D | n.D | n.D | **1.34E+05** | **1.06E+07** | **1.03E+06** | n.D | **1.34E+05** | **2.77E+06** | **5.45E+05** | **2.30E+05** |
| **DSM20339^T^** | **2.14E+05** | n.D | n.D | **1.97E+06** | **2.14E+05** | **1.52E+06** | n.D | n.D | **2.14E+05** | **1.39E+06** | n.D | n.D |
| **Not clearly identifiable** | **1.61E+05** | **7.80E+06** | **2.84E+06** | **3.94E+06** | **1.61E+05** | **4.55E+06** | **4.12E+06** | **2.75E+05** | **1.61E+05** | **1.52E+07** | **2.73E+06** | **4.60E+05** |
| **total CfU/ml** | **2.68E+06** | **1.30E+08** | **7.10E+07** | **9.85E+07** | **2.68E+06** | **1.52E+08** | **1.03E+08** | **2.75E+07** | **2.68E+06** | **1.39E+08** | **5.45E+07** | **2.30E+07** |

Table S2: Cell counts of every strain within the lupine moromi model fermentation in all replicates. The cell counts for every strain were calculated by multiplying the total CfU/ml with the percentual distribution of every strain within a replicate. To calculate the percentual distribution, one hundred colonies were evaluated via colony PCR as described in the materials and methods section. n.D = Not detected; not clearly identifiable = reactions that yield none or more than one band, were not assigned to one specific strain.
